# Receptor Tyrosine Kinase Inhibitor Sunitinib as Novel Immunotherapy to Inhibit Myeloid-Derived Suppressor Cells for Treatment of Endometriosis

**DOI:** 10.3389/fimmu.2021.641206

**Published:** 2021-07-22

**Authors:** Ying He, Sze Wan Hung, Bo Liang, Ruizhe Zhang, Yating Gao, Ching Yan Chu, Tao Zhang, Hui Xu, Jacqueline Pui Wah Chung, Chi Chiu Wang

**Affiliations:** ^1^ Department of Obstetrics & Gynaecology, The Chinese University of Hong Kong, Hong Kong, Hong Kong, SAR China; ^2^ Li Ka Shing Institute of Health Sciences, The Chinese University of Hong Kong, Hong Kong, Hong Kong, SAR China; ^3^ School of Biomedical Sciences, The Chinese University of Hong Kong, Hong Kong, Hong Kong, SAR China; ^4^ Chinese University of Hong Kong–Sichuan University Joint Laboratory in Reproductive Medicine, The Chinese University of Hong Kong, Hong Kong, Hong Kong, SAR China

**Keywords:** endometriosis, myeloid-derived suppressor cells, Sunitinib, immunosuppression, gene expressions

## Abstract

Endometriosis is a common, benign, and hormone-dependent gynaecological disorder that displays altered immunoinflammatory profiles. Myeloid-derived suppressor cells (MDSCs) suppressed immunosurveillance in endometriosis in human and mouse model. Receptor tyrosine kinase inhibitor Sunitinib can induce MDSC apoptosis and suppress the progression of cancer. However, the effects of Sunitinib on MDSCs in endometriosis and the underlying mechanism are not clear. In this study, we employed an animal study of the endometriosis model in mice for treatment of Sunitinib. After syngeneic endometrium transplantation and treatment, endometriotic lesion volume, weight, and histology were compared. Peritoneal fluid, peripheral blood, and bone marrow MDSC subsets and their molecular signaling were monitored by flow cytometry. Peritoneal cytokines were assayed by ELISA. The gene expression profiles of isolated CD11b+Ly6G+Ly6C^lo^ cells were studied by RNA sequencing. We found that Sunitinib significantly decreased the endometriotic lesion size and weight after 1 and 3 weeks, and decreased p-STAT3 activation in MDSCs after 1 week of treatment. In the first week, Sunitinib specifically increased the G-MDSC population in peritoneal fluid but the isolated CD11b+Ly6G+Ly6C^lo^ MDSCs after Sunitinib treatment were presented as mature polynuclear MDSCs, while the control group had immature mononuclear MDSCs. Importantly, we found Sunitinib differentially suppressed gene expressions of immunosuppressive function and differentiation in peritoneal G-MDSCs. Apelin signaling pathway associated genes and inflammation related genes were upregulated, and amino acid metabolism regulator genes were downregulated in bone marrow G-MDSCs. For endometriotic lesions, the PPARG gene governing glucose metabolism and fatty acid storage, which is important for the development of endometriosis was upregulated. In conclusion, Sunitinib inhibited endometriotic lesions, by promoting peritoneal fluid MDSCs maturation and inhibiting the immunosuppressive function. These findings suggest that Sunitinib changed the immune microenvironment and inhibited the development of endometriosis, which has potential therapeutic effects as novel immunotherapy to promote MDSCs maturation, differentiation, and metabolism for the treatment of endometriosis.

## Introduction

Endometriosis is a common, benign, and hormone-dependent gynaecological disorder characterized by the presence of endometrial glands and stroma outside the uterine cavity. It is one of the main causes of pelvic pain, menstrual disorders, and infertility in reproductive women, affecting around 10% of women of reproductive age (1), but it is still under-diagnosed due to the requirement of surgical and/or pathological diagnosis. Currently available treatments are mainly symptom relief, such as hormonal therapies or painkillers; while conservative surgery cannot remove all the endometriosis deposits. Besides the unpleasant hormonal side effects and surgical complications, the recurrence rate of endometriosis after treatment is also high (2).

Endometriosis displays altered immunoinflammatory profiles, which facilitate endometrial tissue to escape immunosurveillance (3). It is hypothesized that women with endometriosis have a defective immune system that is not able to recognize and perform the proper response in defending the endometrial deposits in the ectopic site (4). This promotes the spread of endometrial cells and favors angiogenesis, tissue adhesion, and induces inflammation to promote the growth and development of endometriosis (5).

Myeloid-derived suppressor cells (MDSCs) are a heterogeneous population of myeloid cells that expand under pathological conditions and have immunosuppressive properties by suppressing T cells (6, 7), macrophages (8, 9), and NK cells (10, 11) through multiple mechanisms. Under physiological conditions, bone marrow-derived immature myeloid cells (IMCs) quickly differentiate into monocytic/dendritic progenitor cells (MDP) or myeloblasts (MB) then further develop into macrophages/dendritic cells (DCs) or neutrophils, respectively (12). This differentiation is impaired under tumor microenvironment, infections, or chronic inflammatory conditions, leading to the activation of IMCs and the accumulation of monocytic MDSCs (M-MDSC) and granulocytic MDSCs (G-MDSC) (6). The two subsets of MDSCs use different mechanisms to suppress T cell function and G-MDSC has high levels of reactive oxygen species (ROS); while M-MDSC primarily by nitric oxide (NO), and both subsets have increased arginase 1 (Arg 1). Although G-MDSC is the prominent subtype of MDSCs, M-MDSC has a higher immunosuppressive capacity (13). Previously, our animal study of experimental endometriosis in mice showed that MDSCs were significantly increased in the peritoneal fluid within 24 hours after transplantation of endometrial fragments into the peritoneal cavity, and these cells inhibited T-cell proliferation and expressed high levels of arginase (3). Depletion of MDSCs inhibited the angiogenesis and growth of the endometriotic lesions, indicating MDSCs promote immune escape and the development of endometriosis.

Receptor tyrosine kinase inhibitor Sunitinib is a new target immunomodulator with potent anti-angiogenic and anti-tumor functions. It was approved for anti-cancer treatment, including gastrointestinal stromal tumor and renal cell carcinoma (14). Some clinical studies indicated that Sunitinib treatment promoted a shift from Th-2 to Th-1 response, inhibited Treg cell function (15), and also reduced the numbers of MDSCs in patients with renal cell carcinoma (16). Sunitinib can not only induce MDSC apoptosis and suppress MDSCs immunosuppressive functions *in vitro* but also inhibit Stat3 activity in renal tumor-associated MDSCs (17). Sunitinib showed inhibition of endometriotic lesion growth in rat models (18, 19). But whether Sunitinib specifically targets MDSCs and the effect of Sunitinib on the immune microenvironment of endometriosis still requires further study.

## Materials and Methods

### Mice

Six to seven week old, C57BL/6 female mice were provided by the Laboratory Animal Service Center of the Chinese University of Hong Kong and housed in a pathogen-free animal house at the Prince of Wales Hospital. Mice that weighed 17–22 g were used, 5-10 animals were used in each experiment for analysis. The mice were kept in controlled temperature, humidity, and light conditions. All the mice had free access to food and tap water and were acclimated at least a week before experiments. The study was approved by the Animal Ethics Committee of The Chinese University of Hong Kong.

### Mouse Model of Endometriosis

The endometriosis model was established by surgical transplantation of syngeneic endometrium tissues to the intestinal mesentery (20, 21). Mice were anesthetized by 100mg/kg ketamine and 10mg/kg xylazine before surgery. At 7 days before transplantation (day -7), estrus cycle synchronization was applied by ovariectomy with estrogen supplementation (22). On the day of transplantation (day 0), donor mice were sacrificed and the uterus was dissected and kept in ice-cold sterile 1×PBS. Then the fat tissues around the uterus were removed and a 2 mm biopsy punch was used to obtain the endometrium fragments as implants of equal size. After anesthesia was performed in recipient mice, the abdomen was shaved and disinfected. A small incision around 1 cm was made by small scissors in the middle of the skin and abdominal wall. Then a small section of the intestine was gently pulled out and kept humidified with sterilized PBS. Three implants were carefully sutured to intestinal vessels around 0.5 cm from the bowel with 6-0 black ethilon sutures (Johnson & Johnson, USA, W529H). The bowel was washed with PBS and returned to the peritoneal cavity. The abdominal wall and skin were closed with 5-0 black ethilon suture (Johnson & Johnson, USA, W500H) and disinfected with iodine, then mice were kept on a warm pad for recovery.

### Treatment and Sample Collections

At least 60 mice were randomized to receive either vehicle control (1×PBS) or Sunitinib (40mg/kg/d, LC Laboratories, USA, S-8803) (23) through intraperitoneal daily administration up to 3 weeks from day 0, the behavior and health of mice were monitored every day and body weight was recorded.

Peripheral blood samples were collected *via* orbital sinus on Day 1 before (Day -1), and Day 1 and 7 (or Week 1) after the transplantation. Recipient mice were sacrificed after either 1 week or 3 weeks of treatment for sample collections for various analyses. Peritoneal cells were collected by injecting 3mL ice cold 1×PBS (with 5% fetal bovine serum, FBS) with 2mL air into the peritoneal cavity (24). Bone marrow cells were expelled from the end of the femur bones by flushing with ice-cold PBS using a 1mL syringe with a 25G needle (25). All the cells were centrifuged for 350g 10 mins at 4°C, followed by red blood cell lysis (RBC lysis buffer, Biolegend, USA, 420301) for 10 mins. The cell pellet was resuspended in 5% FBS (Life Technologies, USA, 10270106), in 1×PBS buffer for flow cytometry.

The endometriotic lesions from 10 animals were each removed, then weighed on electronic balance (Sartorius, Germany, N18565). The length and width of the endometriotic lesions were measured by a digital caliper (Mitutoyo, Japan, 50019630, accuracy within 0.01 mm). The endometrial lesion area was calculated using the formula (length×width×π/4) (mm2). The lesions were divided into three parts, one lesion was fixed in 4% formalin then dehydrated in graded ethanol solutions and embedded in paraffin for histological analysis; the other one was snap frozen in liquid nitrogen and stored in -80°C for protein analysis; the last one was immersed in RNA later (Invitrogen Ambion, USA, AM7021) overnight and stored in -80°C for RNA Sequencing.

### Implants Histology by Hematoxylin and Eosin staining

After embedding, 4μm serial paraffin sections of the lesions from five animals were prepared on coated slides. Hematoxylin and Eosin staining were carried out in the middle part of the lesion. Staining procedures were the same as described before (26). After the slides were incubated in a 60°C oven, they were deparaffined in xylene and were hydrated in ethanol at different concentrations (100%, 95%, 80%, 70%). Then slides were stained in hematoxylin, differentiated in acid ethanol, and counterstained with eosin, dehydrated with serial concentrations of ethanol, and then cleared in xylene. Finally, slides were mounted by Dpx (Sigma, USA, 06522) and observed under the microscope.

### Flow Cytometry

Immune cells from at least 10 animals were counted by flow cytometry analysis using FC 500 cytometer (Beckman Coulter, USA). A total of 1×10^6^ cells/100 μL were firstly incubated with anti-mouse CD16/32 (Biolegend, USA, 101320) to block non-specific staining then incubated with antibodies including CD11b - PE/Cy7, Ly-6G - Alexa Fluor 647 (BioLegend, USA, 127610), Ly-6C - FITC (Biolegend, USA, 128006) for MDSCs for 30 mins at 4°C.G-MDSC was defined as CD11b+ Ly6G+ Ly6C^lo^ and M-MDSC was defined as CD11b+ Ly6G-Ly6C^hi^ (3). The fixed cells were permeabilized in pre-chilled True-PhosTM Perm Buffer (Biolegend, USA, 425401) overnight at -20°C, then added intracellular staining antibody anti-STAT3 Phospho (Tyr705) - PE (Biolegend, USA, 651004) for detection of STAT3 Phospho expression as a marker of expansion and migration in MDSCs. The stained cells were washed and resuspended in 1mL staining buffer for further analysis.

### Isolation G-MDSCs

Debris and dead cells were excluded by forward and side scatters gates setting. G-MDSCs from peritoneal fluid and bone marrow from at least 30 animals each were collected and sorted by MoFlo™ Astrios Cell Sorter (Beckman Coulter, USA) by gating as CD11b+ Ly6G+ Ly6C^lo^. After sorting, one part of the isolated cells from 6 animals each was collected for Wright-Giemsa staining, and the other part from another 24 animals each were added with RLT (Qiagen, Germany, 74004) and kept at -80°C for RNA extraction and sequencing.

### Wright-Giemsa Staining

Staining procedures were the same as described in the Wright-Giemsa stain kit (Biovision, USA, K1438-30) according to the manufacturer’s protocol with some modifications. Firstly, two drops of cells were smeared on a clean microscopic slide (Marienfeld, Germany) and dried completely in the hood. Then the slides were fixed in absolute methanol and placed in Wright-Giemsa solution (Biovision, USA, K1438-30) for staining. After that, the slides were rinsed in distilled water and flushed with PBS (pH=6.8). Next, the slides were dipped in distilled water, dried in a hood at room temperature, and cleared in xylene. Finally, slides were mounted and observed under the microscope.

### ELISA

The peritoneal fluid supernatant from at least 10 animals was collected for Enzyme-linked immunosorbent assay (ELISA). Peritoneal fluid cytokines for MDSC recruitment and activation, including Vascular endothelial growth factor (VEGF) (Abcam, UK, ab209882), Granulocyte-colony stimulating factor (G-CSF) (Abcam, UK, ab197743), Interleukin 6 (IL-6) (Abcam, UK, ab222503), Transforming growth factor beta 1 (TGF-β1) (Abcam, UK, ab119557), were measured by ELISA kits. ELISA procedures were conducted following the manufacturer’s instructions. The optical density (OD) was recorded at 450nm by Spectramax Gemini dual-scanning Microplate Spectro (BioTek instrument, USA).

### RNA Extraction and RNA Sequencing

RNA from the endometriotic lesions, isolated bone marrow, and peritoneal fluid MDSCs from 3-4 animals in each treatment group were extracted by RNeasy Mini Kit (Qiagen, Germany, 74104). RNA from the G-MDSC was extracted by RNeasy Micro Kit (Qiagen, Germany, 74004). RNA extraction procedures were followed according to the instructions of the manufacturer. RNA sequencing was performed in the BGISEQ-500 platform and generated more than 20M reads from each sample. The sequencing reads containing low-quality, adaptor-polluted, and high content of unknown base reads were filtered and removed before downstream analyses. After read filtering, clean reads were mapped to the reference genome using HISAT (27). We mapped clean reads to reference transcripts using Bowtie2 (28), then calculated gene expression levels for each sample with RSEM (29). Based on gene expression levels, we defined differentially expressed genes (DEGs) between samples or groups by Deseq2 algorithms with p<0.05 and padj<0.05 or q<0.05. Hierarchical clustering and principal component analysis (PCA) were conducted to show distinct transcriptomic profiles. Gene Ontology classification and functional enrichment were performed with differently expressed genes. KEGG pathway analyses were used to link genes to specific pathways.

### Statistical Analysis

Statistical analysis was carried out with SPSS 21.0 software (IBM, New York, USA). Data were shown as mean ± SEM. Student’s t test was used to compare the statistical changes of numerical data, P<0.05 was determined as significant.

## Results

### Effects of Sunitinib on Development of Endometriotic Lesions

**Figure 1:**
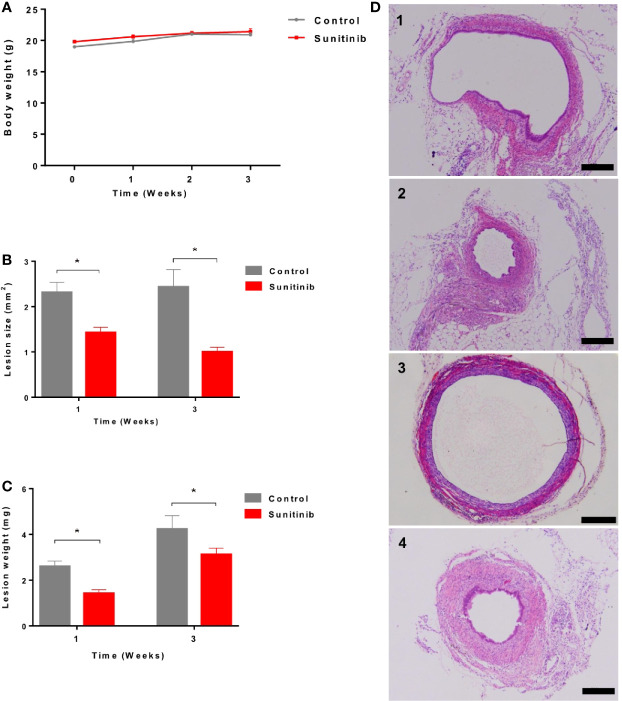
Effects of Sunitinib on endometriosis lesions. **(A)** Changes to body weights during control and Sunitinib treatment. **(B)** Size of the endometriotic lesions after 1 and 3 weeks of treatment. **(C)** Weight of the endometriotic lesions after 1 and 3 weeks of treatment. Data are expressed as the mean ± SEM from 10 animals in each group, *p < 0.05. **(D)** Representative histology sections of the endometriotic lesions from control and Sunitinib groups. D1: endometriotic lesion after 1 week of control treatment; D2: endometriotic lesion after 1 week of Sunitinib treatment; D3: endometriotic lesion after 3 weeks of control treatment; D4: endometriotic lesion after 3 weeks of Sunitinib treatment, Scale bar: 200μm.

All the animals tolerated the treatment well and showed no significant change in body weight during the treatment (**Figure 1A**). Compared to the control group, endometriotic lesion size and weight were significantly decreased after 1 week and 3 weeks of Sunitinib treatment (**Figures 1B, C**). Endometrial glandular tissue was confirmed in all the implanted endometriotic lesions in both groups (**Figure 1D**). Compared to the control group, endometrial glands in the Sunitinib group were under-developed with small endometriotic sac in both week 1 and week 3 of the treatment.

### Effects of Sunitinib on MDSCs and Peritoneal Cytokines

**Figure 2:**
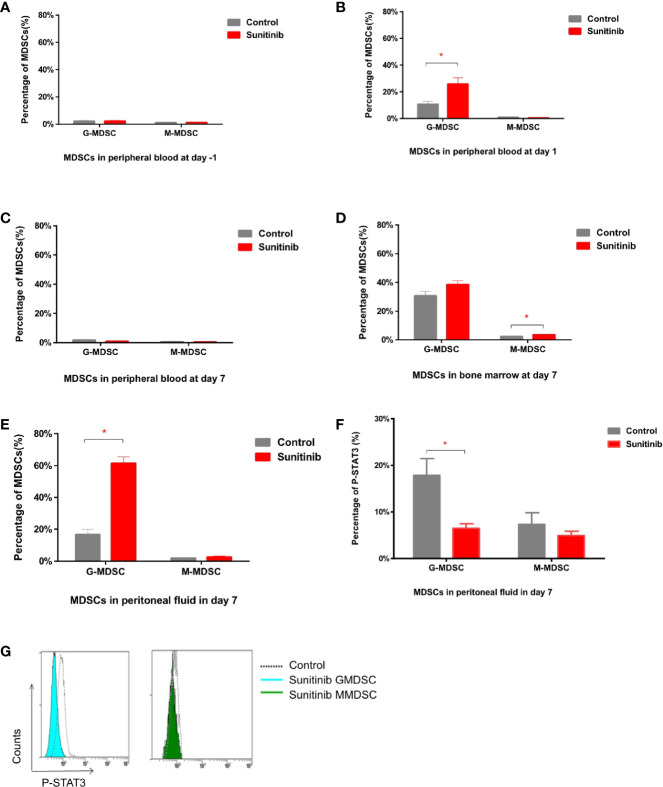
MDSC subsets in peritoneal blood, bone marrow, and peripheral fluid after Sunitinib treatment. **(A–C)** G-MDSC (defined as CD11b^+^ Ly6G^+^ Ly6C^lo^) and M-MDSC (defined as CD11b^+^ Ly6G^-^ Ly6C^hi^) percentage in peripheral blood 1 day before as baseline (day -1), after 1 day (day 1) and 7 days (day 7) of control and Sunitinib treatment. **(D, E)** G-MDSC and M-MDSC percentage in the bone marrow and peritoneal fluid after 7 days of treatment. **(F)** Percentage of P-STAT3 in peritoneal G-MDSC and M-MDSC after 7 days of treatment. **(G)** Representative flow cytometric analyses of intracellular levels of p-Stat3 in peritoneal MDSCs. Data are expressed as the mean ± SEM from 5 animals in each group, *p < 0.05.

In peripheral blood, G-MDSC and M-MDSC populations were very low and showed no significant difference between groups in baseline 1 day before the Sunitinib and vehicle treatment (**Figure 2A**). After 1 day of treatment, G-MDSC was significantly increased in the Sunitinib group when compared with control, M-MDSC remained low and no significant change (**Figure 2B**). However the increase of G-MDSC disappeared and became non-significant in the Sunitinib group, M-MDSC remained low and no significant change was observed after 7 days of treatment (**Figure 2C**). In the bone marrow, G-MDSC were slightly increased in the Sunitinib group, but there was no significant difference from the control group after 7 days of treatment. Whilst M-MDSC were significantly increased in the Sunitinib group when compared with the control (**Figure 2D**). In peritoneal fluid, G-MDSC were significantly increased in the Sunitinib group when compared with control, but M-MDSC remained low and there was no significant change after 7 days of treatment (**Figure 2E**). Taken together, Sunitinib affected the G-MDSC population more than the M-MDSC population.

**Figure 3:**
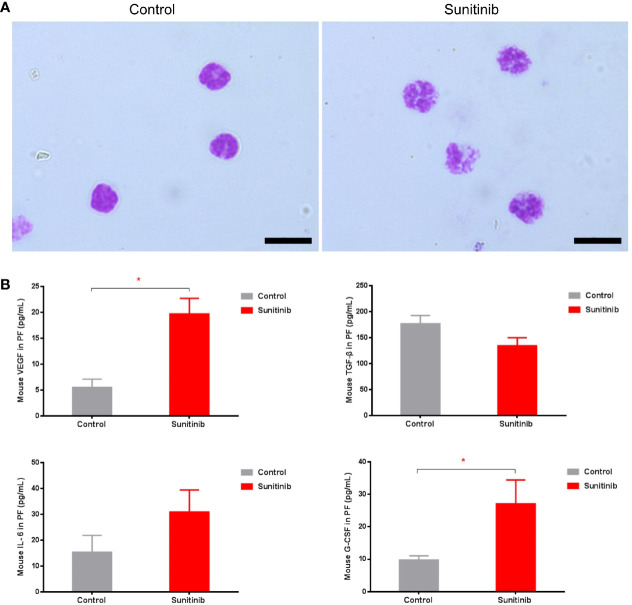
Characteristics of G-MDSC cells and peritoneal cytokine after Sunitinib treatment. **(A)** Representative Wright-Giemsa staining of isolated peritoneal CD11b+ Ly6G+ G-MDSCs from at least 5 animals in each group after 7 days of treatment, Magnification 1000× Oil, and Scale bar 10μm. **(B)** Peritoneal cytokines VEGF, G-CSF, IL-6, TGF-β after 7 days of treatment. Data are expressed as the mean ± SEM from 10 animals in each group, *P < 0.05.

Moreover, phospho-STAT3 was significantly reduced in the G-MDSC, but not the M-MDSC, in the Sunitinib group compared with control after 7 days of treatment (**Figures 2F, G**). The isolated CD11b+Ly6G+ Ly6C^lo^ G-MDSC in the control group were all mononuclear cells, while those in the Sunitinib group were all polynuclear cells (**Figure 3A**). In peritoneal fluid, VEGF and G-CSF concentrations were significantly increased in the Sunitinib group when compared with control (**Figure 3B**). While IL-6 concentrations were slightly increased and TGF-β concentrations were slightly decreased in the Sunitinib group, but there were no significant differences between groups.

### Effects of Sunitinib on Gene Expression of G-MDSC

**Figure 4:**
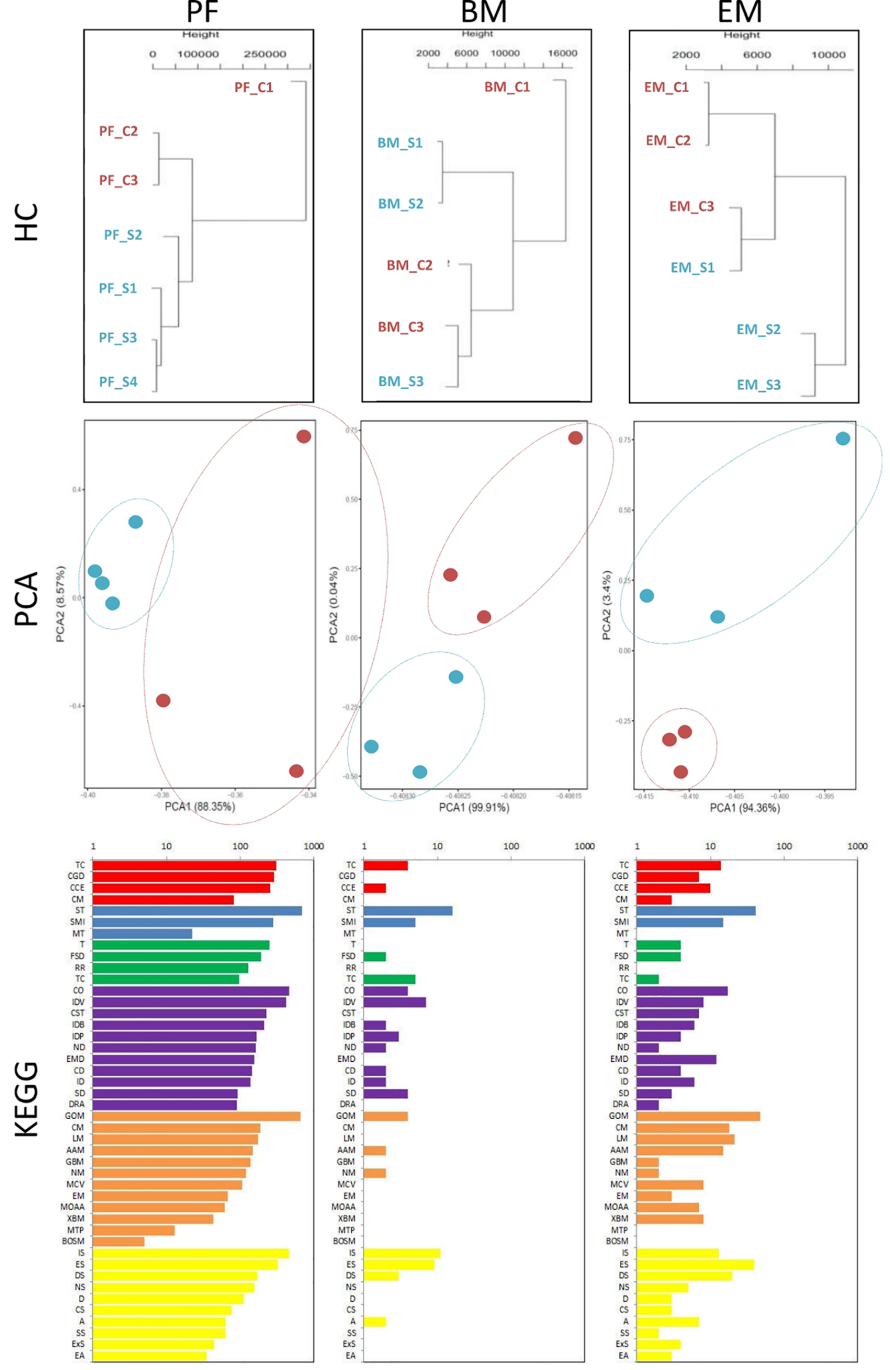
Transcriptomic characteristics of G-MDSC cells and endometriotic lesions. (Upper and middle panels) Hierarchical clustering (HC) and principal component analysis (PCA) of peritoneal (PF) and bone marrow (BM) CD11b+ Ly6G+ G-MDSCs and endometriosis lesions (EM) from 3-4 animals in each group after 7 days of Sunitinib treatment (S in blue labels and dots) versus control (C in red labels and dots). For HC, the closer the samples were to each other, the more similar the expression level was. For PCA, the first and second most coordinated components are shown, similar transcriptomic profiles were grouped by circles. (Lower panels) KEGG Pathway functional enrichment. X axis represents the number of DEG, Y axis represents the functional classification of KEGG according to 7 branches for KEGG pathways: Cellular Processes (red), Environmental Information Processing (blue), Genetic Information Processing (green), Human Disease (purple), Metabolism (orange), and Organismal Systems (yellow). TC, Transport and catabolism; CGD, Cell growth and death; CCE, Cellular community—eukaryotes; CM, Cell motility; ST, Signal transduction; SMI, Signaling molecules and interaction; MT, membrane transport; T, translation; TS, transcription; CO, Cancer—overview; IDV, infectious diseases—viral; CST, Cancers—specific types; IDB, infectious diseases—bacterial; IDP; infectious diseases—parasitic; ND, neurodegenerative diseases; EMD, endocrine and metabolic diseases; CD, cardiovascular diseases; ID, Immune diseases; SD, substance dependence; DRA, drug resistance—antineoplastic; GOM, Global and overview maps; CM, carbohydrate metabolism; LM, Lipd metabolism; AAM, Amnio acid metabolism; Glycan biosynthesis and metabolism; NM, nucleotide metabolism; MCV, Metabolism of cofactors and vitamins; EM, energy metabolism; MOAA, Metabolism of other amnio acids; XBM, Xenobiotics biodegradation and metabolism; MTP, Metabolism of tepenoids and polyketides; BOSM, Biosynthesis of other secondary metabolires; IS, Immune system; ES, Endocrine system; DS, Digestive system; NS, Nervous system; D, Development; CS, Cirrculatory system; A, Aging; SS, Sensory system; ExS, Excretory system; EA, Environmental adaptation.

We collected peritoneal and bone marrow G-MDSC and endometriotic lesions after Sunitinib and control treatment for RNA-seq, but only 3-4 samples in each tissue type had sufficient G-MDSC with high-quality RNA for the analysis. Although the sample size was small, hierarchical clustering and principal component analysis showed distinct transcriptomic profiles in the Sunitinib group from the control group (**Figure 4**). Based on KEGG analysis, Sunitinib had more profound effects on the biological processes, gene functions, and associated diseases in peritoneal G-MDSC than that in bone marrow G-MDSC and endometriotic lesions, while bone marrow G-MDSC had minimal effects when compared with other samples.

### Peritoneal G-MDSC

**Figure 5:**
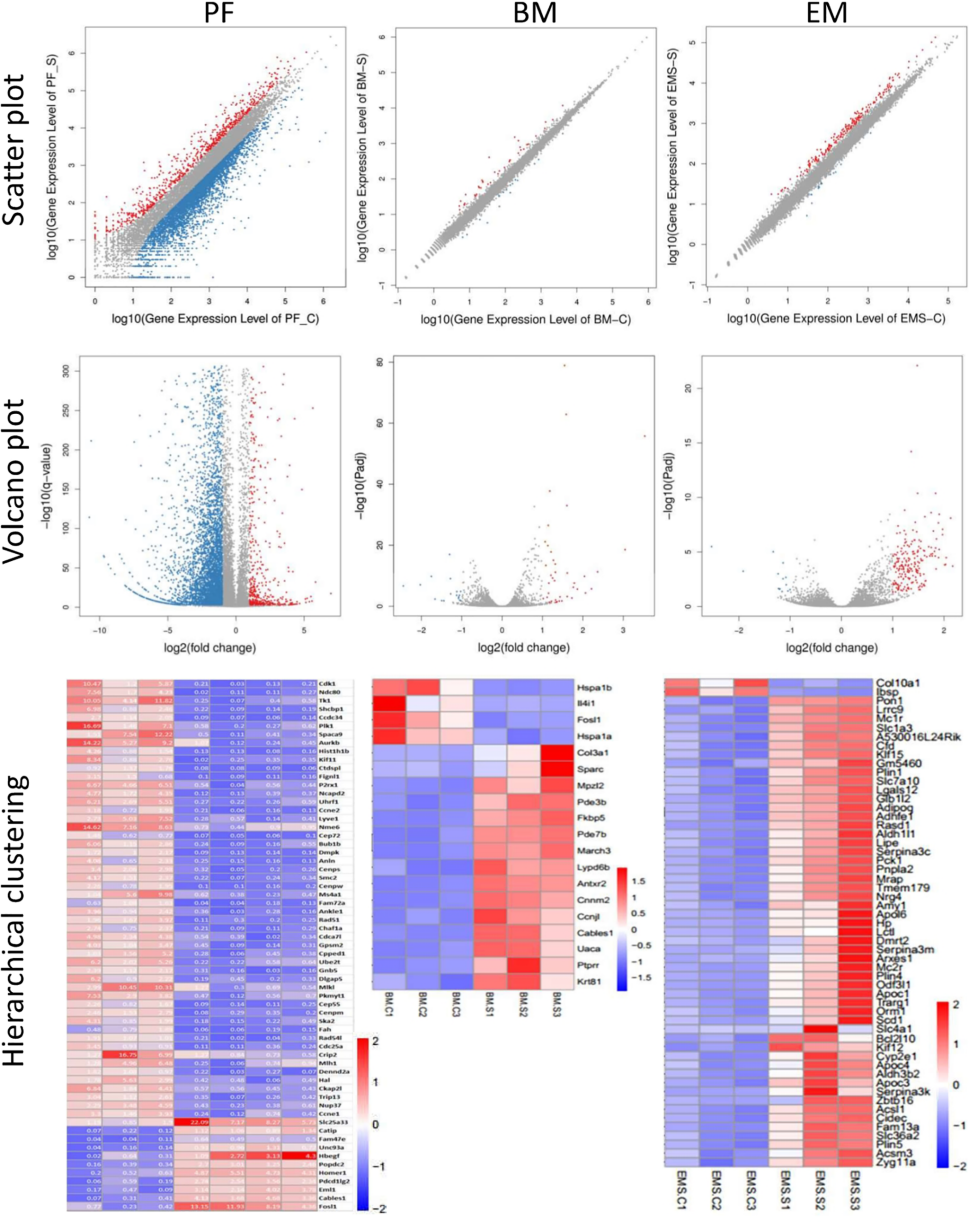
Differential gene expression of G-MDSC cells and endometriotic lesions. (Upper panels) Scatter plots of peritoneal (PF) and bone marrow (BM) CD11b+ Ly6G+ G-MDSCs and endometriosis lesions (EM) from 3-4 animals in each group after 7 days of Sunitinib treatment (S) versus control (C). X Y axis represents log10 transformed gene expression level, red color represents the up-regulated genes, the blue color represents the down-regulated genes, gray color represents the non-DEGs. (Middle panels) Volcano plots of peritoneal (PF) and bone marrow (BM) CD11b+ Ly6G+ G-MDSCs and endometriosis lesions (EM) from 3-4 animals in each group after 7 days of Sunitinib treatment (S) *versus* control (C). X axis represents log2 transformed fold change. Y axis represents -log10 transformed significance. Red points represent up-regulated DEGs. Blue points represent down-regulated DEGs. Gray points represent non-DEGs. (Lower panels) Heat map of peritoneal (PF) and bone marrow (BM) CD11b+ Ly6G+ G-MDSCs and endometriosis lesions (EM) from 3-4 animals in each group after 7 days of Sunitinib treatment (S) *versus* control (C) by hierarchical clustering. X axis represents the sample. Y axis represents the DEGs. The color represents the log_2_ transformed gene expression level. The dark color means the high expression level while the light color means the low expression level. The most significant differentially expressed genes (p < 0.05, padj < 0.05 or q < 0.01, and also fold changes > 1.5-5) are shown. The full list and statistical analysis are available in corresponding **Supplementary Tables 1**–**3**.

5748 genes were differentially expressed with p<0.05, padj<0.05 or q<0.01 in peritoneal G-MDSCs after Sunitinib treatment, while only 62 genes with further fold changes had >5 (**Figure 5** and **Supplementary Table 1**). Only 721 genes were significantly up-regulated and 5027 genes were significantly down-regulated.

#### Granules Proteins

Amongst all genes, primary granules genes, including *Myeloperoxidase (MPO), Bactericidal Permeability Increasing Protein (BPI), Proteinase 3 (PRTN3)*, and *Cathepsin G (CTSG)*, were significantly decreased in the Sunitinib group. Secondary granules genes, including *Matrix Metalloproteinase 8* (*MMP8), Cytochrome b-245, beta Polypeptide (CYBB), Lipocalin 2 (LCN2), Stomatin (STOM), Cathelicidin Antimicrobial Peptide (CAMP), Carcinoembryonic Antigen-related Cell Adhesion Molecule 1 (CEACAM1)*, and *Lactotransferrin (LTF)*, were decreased in the Sunitinib group. While tertiary granules gene, *Peptidoglycan Recognition Protein 2 (PGLYRP2)* was significantly down-regulated in the Sunitinib group.

#### Transcription Factors

Granulocyte differentiation regulator genes, CCAAT enhancer-binding protein alpha (CEBPA), and epsilon (CEBPE), were decreased, and CCAAT enhancer-binding protein beta (CEBPB) and zeta (CEBPZ) were increased.

#### Phagocytosis

Many genes associated with phagosome formation and maturation were also differently expressed. *Membrane related protein SEC61, gamma Subunit (SEC61G)* was significantly decreased in the Sunitinib group. Phagocytic associated enzymes, such as *PTK2 Protein Tyrosine Kinase 2 (PTK2), Phospholipase D2 (PLD2*), and *Cathepsin*, were decreased in the Sunitinib group. While *Triggering Receptor Expressed on Myeloid Cells 1 (TREM1)* was significantly up-regulated in the Sunitinib group.

#### Immune Suppression

ARG1 and NOS2 were significantly down-regulated in the Sunitinib group compared with the control. TGF- β2 was also significantly down-regulated in the Sunitinib group.

#### Other Changes

Genes that have an important role in microorganism recognition, such as *Toll-like Receptor 2 and 4* (*TLR2 and TLR4)*, were significantly up-regulated in the Sunitinib group.

### Bone Marrow G-MDSC

Only 56 genes were differentially expressed in bone marrow G-MDSC after Sunitinib treatment (**Figure 5** and **Supplementary Table 2**). In total, 39 genes were significantly up-regulated and 17 genes were significantly down-regulated. Amongst all genes, increased expression of Apelin signaling pathway associated genes, including *Phosphoinositide-3-kinase Interacting Protein 1 (PIK3IP1), Secreted Phosphoprotein 1 (SPP1)*, and *Phosphodiesterase 3B, cGMP-inhibited (PDE3B)*, were found. Genes related to cell adhesion molecules, including *Myelin Protein Zero-like 2 (MPXL2) and Programmed Cell Death 1 Ligand 2 (PDCD1LG2)*, were up-regulated. *Acyl-CoA Thioesterase 1 (ACOT1)* took part in the biosynthesis of unsaturated fatty acids and was up-regulated. *Interleukin 1 Receptor Accessory Protein (IL1RAP)*, which is involved in the MAPK signaling pathway, was up-regulated. While *Interleukin 4Iinduced 1 (IL4I1)* involved in alanine, aspartate and glutamate metabolism, phenylalanine, tyrosine, and tryptophan biosynthesis, phenylalanine metabolism, tyrosine metabolism, were all down-regulated after Sunitinib treatment.

### Endometriotic Lesions

In total, 219 genes were differentially expressed in endometriotic lesions after Sunitinib treatment (**Figure 5** and **Supplementary Table 3**). 205 genes were significantly up-regulated but only 14 genes were significantly down-regulated. Based on KEGG pathway databases, the most enriched up-regulated pathways included metabolism, PPAR signaling, and AMPK signaling pathways, while down-regulated pathways included the cAMP signaling pathway. For the PPAR signaling and metabolism pathways, *Phosphoenolpyruvate Carboxykinase 1, Cytosolic (PCK1), Acyl-Coenzyme A Oxidase 1, Palmitoyl (ACOX1), Acyl-CoA Synthetase Long-chain Family Member 1 (ACSL1*); and *Acetyl-Coenzyme A Acyltransferase 1B (ACAA1B)* were up-regulated. While *Stearoyl-Coenzyme A Desaturase 1 (SCD1)* and *Peroxisome Proliferator Activated Receptor Gamma (PPARG)* were up-regulated.

## Discussion

In our study, we demonstrated that Sunitinib significantly inhibits endometriosis growth and development after one week of treatment, indicating that Sunitinib interferes with the development of endometriosis in the early stage. Here we showed that Sunitinib modulated immune response by inhibiting MDSCs, contributing to the suppression of endometriosis development.

MDSCs are a population of immature myeloid cells that negatively regulate immune responses in pathological conditions, such as cancer and other diseases (6). G-MDSC account for a major proportion of MDSCs, while M-MDSC can differentiate into dendritic cells and macrophages. An increased number and proportion of G-MDSC was observed *in vitro* MDSCs culture, which suggested that M-MDSC can be differentiated into G-MDSC (30). The increased M-MDSC in bone marrow might lead to higher G-MDSC in peripheral blood, then elevated G-MDSC in peritoneal fluid. In our previous study, we demonstrated that MDSCs promoted immune escape and the development of endometriosis (3). Although we cannot confirm whether G-MDSC or M-MDSC is responsible for the development of endometriosis, it is most likely to be G-MDSC since these cell populations are more prominent in both women with endometriosis and mice of endometriosis model (3). The present study indicated that Sunitinib may induce MDSCs expansion from the bone marrow into blood and finally migrate into the peritoneal cavity in endometriosis. On the other hand, we also found that CD11b+ Ly6G+ G-MDSCs in the control group were all mononuclear, i.e. more pro-endometriotic, while the G-MDSC in the Sunitinib group were polynuclear, i.e. more anti-endometriotic. In a tumor mouse model, TGF-β blockade turned the CD11b+Ly6G+ circular nuclei pro-tumor neutrophils into hypersegmented anti-tumor neutrophils, along with increased CD8+T cell activation (31). These morphological characteristics may suggest that the CD11b+Ly6G+ cells in the Sunitinib group are anti-tumor N1 neutrophils while they remain pro-tumor N2 neutrophils or immunosuppressive G-MDSC in the absence of Sunitinib. Although we did not perform functional neutrophil assays and CD8+ T cell activation to confirm, our RNA-seq data in the isolated peritoneal G-MDSC indicated the direction of the immune activation (**Supplementary Table 1**).

Moreover, we found peritoneal CD11b+ Ly6G+ G-MDSC was significantly increased but p-stat3 was decreased in the Sunitinib group. Activation of STAT3 promotes tumor proliferation, angiogenesis, and metastasis, facilitates pro-tumor inflammation, and inhibits Th1 anti-tumor immune responses (32). Our study indicated that decreased p-stat3 may suggest that these G-MDSC have lower immune suppressive activity after treatment of Sunitinib. While cytokines IL-6, VEGF, G-CSF can promote the activation of STAT3, which in turn contributes to the proliferation and survival of immature myeloid cells and prevents their maturation (33). TGF-β is a key cytokine to induce N2 pro-tumor neutrophil production within tumors and also take part in the suppressive mechanism of MDSCs. In our study, Sunitinib treatment for one week significantly increases VEGF and G-CSF levels compared with the control group. The increased VEGF levels in peritoneal fluid might result from drug induced suppression in the vascular function of endometriotic lesions in the early development of endometriosis. Whilst G-CSF can mobilize hemopoietic stem cells from the bone marrow into the blood and promotes neutrophil lineage differentiation (34). In our study, elevated G-CSF in peritoneal fluid corresponded with an increase in G-MDSC in peritoneal fluid and peripheral blood, suggesting potential neutrophil differentiation after Sunitinib treatment. Our RNA-seq data also showed pro-angiogenesis molecules, including C-C motif chemokine 22 precursors, forkhead box protein P3 isoform X1, collagen alpha-1(XXI) chain, and angiopoietin-related protein 1 precursor were suppressed in the lesions (**Supplementary Table 3**).

Several key factors are implicated in the immune suppression of G-MDSC including arginase 1 (ARG1), iNOS, reactive oxygen species (ROS), and peroxynitrite (PNT) (6). In G-MDSC from peritoneum or spleen of the tumor mouse model, increased ARG 1 and MPO activity, higher ROS production, lower phagocytosis associated enzymes were found when compared to neutrophils from tumor-free mice (35). In our study, significantly decreased ARG 1 and MPO activity in peritoneal CD11b+Ly6G+ cells after Sunitinib treatment was found, which indicated that these cells have lower immune suppressive activity. TGF-β2 was significantly down-regulated in peritoneal CD11b+Ly6G+ cells after Sunitinib treatment. While TGF-β blockade promotes the production of N1 neutrophils with antitumor activity (31), it suggests that Sunitinib treatment may increase the production of N1 neutrophils. While Stat3 increases the survival of MDSCs by promoting the expression of myelocytomatosis oncogene (MYC), cyclin D1 (Ccnd1) (6), in our study Sunitinib treated peritoneal CD11b+Ly6G+ cells express significantly lower levels of MYC, Ccnd1 demonstrate the decreased activity of Stat3 after treatment.

However, the relationship between G-MDSC and neutrophils remains unclear, although they share similar surface phenotypes and granulocyte morphology. Identifying the molecular and gene profile changes in G-MDSC and endometriotic lesions after Sunitinib treatment will shed light on the potential mechanism of anti-endometriosis functions. During granulocytic differentiation, the primary, secondary, and tertiary granules and granule proteins are formed at different stages during maturation. In our study, primary, secondary and tertiary granule expression were lower in peritoneal CD11b+Ly6G+ cells after Sunitinib treatment, this suggests that these cells become more mature after treatment. CCAAT/enhancer binding proteins (C/EBPs) are transcription factors that regulate granule protein expression and the differentiation of many cell types, control cell cycle and cell proliferation (36). CEBPA, CEBPB, and CEBPE are the most widely studied isoforms of the C/EBP family, while CEBPA and CEBPE are the key regulators that govern differentiation of hematopoietic stem cells (HSCs) along the myeloid lineage toward granulocytes rather than monocytes (37). CEBPA is necessary for the differentiation of neutrophil precursors to myeloblasts, knock-out of CEBPA in mice results in a block of granulocytic differentiation and the complete absence of mature granulocytes (38). CEBPB and CEBPZ increase during granulopoiesis is abundantly expressed on mature cells (39). CEBPE is mainly found in myelocytes and metamyelocytes but is rarely expressed in more mature cells (39). In our study, *CEBPA* and *CEBPE* expression was decreased, and *CEBPB* and *CEBPZ* expression was increased, it also indicated peritoneal CD11b+Ly6G+ cells after Sunitinib treatment become more mature.

Different from the peritoneal fluid and bone marrow, Sunitinib interferes with lipid metabolism in endometriotic lesions. Lipid metabolism has been proposed to play an important role in the growth and development of endometriosis. PPARs are crucial regulators of cellular differentiation, development, tumorigenesis, metabolism, inflammation (40). PPARγ agonists can not only modulate glucose and lipid metabolism but also have anti-inflammatory effects, in an experimental endometriosis rat model, PPARγ agonists rosigliotazone treatment or no medication control were given for 4 weeks, the size and weight of endometriotic lesions were significantly decreased in rosiglitazone-treated groups compared with the control (41). This indicates that Sunitinib may act as PPARγ agonists, modulating lipid metabolism and inflammation and improve insulin sensitivity to suppress endometriosis development. Further study is required to confirm the underlying mechanism.

Although Sunitinib seems to be quite safe, it has been occasionally associated with potentially severe adverse reactions in treating cancer patients, including, hypertension, hepatotoxicity (42), and, less frequently, posterior encephalopathy (43), cardiotoxicity (44), and hepatotoxicity (45). Whether the side effects were associated with the medications or health conditions still cannot be determined. Future animal studies or clinical trials of endometriosis, blood pressure, blood picture, and brain, cardiac, and liver functions should also be monitored. On the other hand, Sunitinib has been shown to induce epithelial to mesenchymal transition and accelerate motility cancer cells (46). However, in our RNA-seq data, we did not find any significant changes in transcripts associated with gap junctions, cadherin, vimention, integrins, and cytoskeleton molecules (**Supplementary Tables 1**–**3**).

## Conclusion

Sunitinib inhibited endometriotic lesion by promoting peritoneal fluid myeloid-derived suppressor cells (MDSCs) maturation and inhibiting its immunosuppressive functions. Sunitinib changed the immune microenvironment and inhibited the development of endometriosis, which has potential therapeutic effects as novel immunotherapy to promote MDSCs maturation, differentiation, and metabolism for the treatment of endometriosis.

## Data Availability Statement

The accession number for the processed expression data of RNA-seq reported in this paper is PRJNA625083 (https://www.ncbi.nlm.nih.gov/Traces/study/?acc=PRJNA625083).

## Ethics Statement

The animal study was reviewed and approved by the government of the Hong Kong Special Administrative Region Department of Health.

## Author Contributions

YH and CCW designed the study. YH, SWH, BL, RZ, YG, CYC performed the experiments. YH wrote the manuscript. TZ, HX, PWC and CCW revised the article. All authors reviewed and approved the submission of the article.

## Funding

This research was supported by Health & Medical Research Fund (08190886) from Food and Health Buerau; The Hong Kong Obstetrical & Gynaecological Trust Fund 2019 from Hong Kong Society of Obstericians and Gynaecologists; Academic Equipment Grant (3029876); and Direct Grant (2017.044) from The Chinese University of Hong Kong.

## Conflict of Interest

The authors declare that the research was conducted in the absence of any commercial or financial relationships that could be construed as a potential conflict of interest.

## Supplementary Material

Supplementary Table 1 Differential expression genes (DEG) of peritoneal G-MDSC.

Supplementary Table 2 Differential expression genes (DEG) of bone marrow G-MDSC.

Supplementary Table 3 Differential expression genes (DEG) of endometriotic lesions.
