# Correction: Receptor tyrosine kinase inhibitor sunitinib as novel immunotherapy to inhibit myeloid-derived suppressor cells for treatment of endometriosis

**DOI:** 10.3389/fimmu.2025.1741271

**Published:** 2025-11-24

**Authors:** Ying He, Sze Wan Hung, Bo Liang, Ruizhe Zhang, Yating Gao, Ching Yan Chu, Tao Zhang, Hui Xu, Jacqueline Pui Wah Chung, Chi Chiu Wang

**Affiliations:** 1 Department of Obstetrics & Gynaecology, The Chinese University of Hong Kong, Hong Kong, Hong Kong SAR, China; 2 Li Ka Shing Institute of Health Sciences, The Chinese University of Hong Kong, Hong Kong, Hong Kong SAR, China; 3 School of Biomedical Sciences, The Chinese University of Hong Kong, Hong Kong, Hong Kong SAR, China; 4 Chinese University of Hong Kong–Sichuan University Joint Laboratory in Reproductive Medicine, The Chinese University of Hong Kong, Hong Kong, Hong Kong SAR, China

**Keywords:** endometriosis, myeloid-derived suppressor cells, Sunitinib, immunosuppression, gene expressions

An incorrect **Funding** statement was provided. The correct **Funding** statement reads:

“This research was supported by The Hong Kong Obstetrical & Gynaecological Trust Fund 2019 from Hong Kong Society of Obstericians and Gynaecologists; Academic Equipment Grant (3029876); and Direct Grant (2017.044) from The Chinese University of Hong Kong.”

The original version of this article has been updated.

